# Poisoning by the Berries of Rhamnus Frangula

**Published:** 1885-12

**Authors:** 


### POISONING BY THE BERRIES OF RHAMNUS FRANGULA.

Dr. O. Petersen reports in the St. Petersburg *Nieue Wocherschrift*, No. 37, 1885, a case of poisoning, and remarks: "In general the berries of rhamnus frangulæ are considered harmless, and the lay-folks use it frequently for diarrhœa."

July 29th the doctor was called hastily in the evening to the son of a farmer who was unconscious and in convulsions. It was harvest time. The boy accompanied his father over the fields, and about four P. M. ate some berries of rhamnus. Driving home about eight, he complained of headache and vertigo. At home he wanted to moisten his head in the waters of a creek, but lost his balance, fell into the water, but was immediately removed from it. The vertigo steadily increased, consciousness failed, he began to act furiously, and complained of excruciating pains.

About half-past nine Petersen found the well-nourished boy of eleven years lying near the porch, thrashing about in such a manner that three persons could hardly keep him down. He screamed and laughed, and there were spasmodic twitchings of the muscles of mastication. The extremities twitched constantly; no opisthotonos. There was no regularity in the spasms, but chronic spasms prevailed. Consciousness was totally abolished, the pupils dilated equally on both sides, with slow and trifling reaction. Pulse greatly accelerated, small, filiform. The doctor tried to raise him up in order to give him some wine, but the muscles of deglutition failed to act and most of it ran out of his mouth. After repeated cold douches the pulse became stronger, the respiration rose, and the boy made some motions showing his dislike to the cold affusions. The spasms decreased so that a clysma could be given, which was retained, as well as a second one, only the gurgling of the fluid in the large intestines could be heard, showing intestinal paralysis, though the sphincter was still acting. The affusions were continued at longer intervals, ether given internally, and after three-quarters of an hour consciousness slowly returned, a copious alvine discharge followed, containing with much fœcal matter the black skins and pits of the berries. Copious draughts of hot water and every ten minutes two teaspoonfuls of Vinum Ipecacuanhæ caused copious vomiting and discharge of berries and pits. He soon fell asleep and the next morning complained only of weakness, pains in the pit of the stomach, and a heavy, white-coated tongue heated to acute gastric catarrh. After a few days he felt perfectly well. It seems that the poison—hydrocyanic acid—is not so much contained in the berries as in the pits, which in this case the boy masticated.

—

Allen, Vol. VIII, page 302, has a short article on rhamnus frangula, black alder, blackthorn alder (which he translates Zrech-wegdorn, whereas Petersen says Faulbaum), of which the texture and triturations of the bark, gathered in spring from the younger branches, is used. We read here: Vertigo, dullness of head, frontal headache, increased peristaltic movements of the bowels, rumbling in abdomen, profuse emission of flatus, accelerated pulse, general exhaustion, weakness of all the limbs, great distress.

The *United States Dispensatory*, fifteenth edition, page 698, says of it: "In its fresh state this drug is very irritant to the gastro-intestinal mucous membrane, producing, when taken in sufficient quantity, violent catharsis, accompanied by vomiting and much pain."

Bartholow, Wood, Hale do not mention it. Phillips (*Mat. Med. and Therapeutics*, 105) considers it a hydragogue cathartic and vermifuge (sometimes emetic), and well adapted to some cases of habitual constipation. *Its vermifuge properties should not be overlooked.*

This last sentence made me compare the symptoms of Rhamnus frangala with Santonine, as I saw a fatal case of poisoning with the latter, and showing similar symptoms, as unconsciousness, vertigo, dilated pupils, convulsive movements of the muscles of the face, especially of the lips and lids, nausea and vomiting, rumbling in abdomen, respiration rapid, pulse feeble and quick, most violent convulsions, with loss of consciousness, chronic spasms, beginning in face and extending downward, collapse.

Every case of poisoning is only a verification of our provings and will thus enlarge our knowledge of materia medica.

